# FEASIBILITY OF A LOW-DOSE FRAILTY PREVENTION INTERVENTION AMONG OLDER AFRICAN AMERICANS

**DOI:** 10.1093/geroni/igz038.1079

**Published:** 2019-11-08

**Authors:** Heather A Fritz, Wassim Tarraf, Pragnesh Patel

**Affiliations:** Wayne State University, Detroit, Michigan, United States

## Abstract

Older African Americans (OAA) are at high risk for becoming frail in later life. Interventions can reverse or delay frailty, yet OAA have largely been excluded from frailty intervention research. Many interventions are also time and resource intensive, making them inaccessible to socially disadvantaged OAA. We present results of a feasibility trial of a low dose frailty prevention intervention among 60 community-dwelling, pre-frail OAA aged 55+ recruited from a primary care clinic between June 1st and October 31st 2018. Using a 2-arm RCT, participants were assigned to the intervention, which was delivered by an occupational therapist (OT) and comprised of four sessions over four months (an OT evaluation, and sessions on healthy dietary practices, increasing physical activity, and maintaining a healthy lifestyle), or enhanced usual care (publicly available information about healthy lifestyle, home safety, and local elder services). Feasibility criteria were set a priori at 75% for participant retention (including attrition due to death/hospitalization), 80% for session engagement, 2 participants/week for mean participant accrual, and 90% for program satisfaction. Participants were 65% female with an average age of 76.58 years, 51.67% of which lived alone and 51.67% lived off of less than 15K per year. Feasibility metrics were met. The study recruited 2.5 participants per week and retained 75% of participants who attended 95% of scheduled sessions. Mean satisfaction scores were 93%. The intervention was feasible to deliver. Qualitative findings from exit interviews suggested changes to the program dose, structure, and content that could improve it for future use.

